# Potassium‐Dependent Coupling of Retinal Astrocyte Light Response to Müller Glia

**DOI:** 10.1002/glia.70022

**Published:** 2025-04-22

**Authors:** Joseph Matthew Holden, Andrew M. Boal, Lauren Katie Wareham, David John Calkins

**Affiliations:** ^1^ Department of Ophthalmology and Visual Sciences Vanderbilt University Medical Center Nashville Tennessee USA; ^2^ Vanderbilt Neuroscience Graduate Program Vanderbilt University Nashville Tennessee USA; ^3^ University of Michigan Ann Arbor Michigan USA

**Keywords:** astrocyte, electrophysiology, light, Müller glia, potassium, retina

## Abstract

Astrocytes throughout the central nervous system mediate a variety of functions to support proper tissue physiology, including the regulation of blood flow and providing metabolic support to neurons. There is also growing appreciation for their role in directly modulating neuronal excitability and information transfer. Recently, we reported that astrocytes in the retina exhibit an array of neuronal‐associated microstructural motifs whose structure and placement suggest roles in monitoring neuronal electrical activity or direct modulation of excitability. In this study, we record whole‐cell patch clamp responses of astrocytes in intact retina to both light and voltage step as a precursor to studying the detailed physiology of individual microstructural motifs. Retinal astrocytes exhibit small amplitude, graded depolarization to both light ON and OFF stimuli with waveforms that closely resemble those of Müller glial endfeet, from which we also recorded. Depolarization is due to potassium influx, with the major source likely being focal release from Müller endfeet onto astrocyte soma. Both macroglia additionally share current–voltage relationships and exhibit stimulus‐dependent changes in ionic permeability. The results suggest a pathway of communication from Müller cells to astrocytes that could support broader retinal modulation beyond potassium spatial buffering.

## Main Points

- Astrocytes depolarize to light ON and OFF like Müller glia.
- The astrocyte light response is due to potassium influx.
- Retinal macroglial current‐voltage responses exhibit rectification which changes with repeated stimulation.

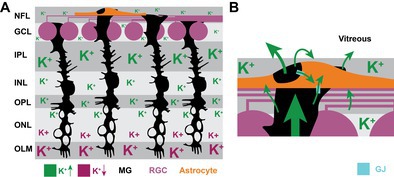

## 1 Introduction

Astrocytes are the predominant macroglia cell of the central nervous system (CNS), in some estimates outnumbering neurons in their resident tissues (von Bartheld et al. 2016). They serve diverse functions, including the regulation of blood flow, maintaining ionic balance, providing metabolic support to neurons, and restoring homeostatic conditions following insult (Macvicar and Newman 2015; Roumes et al. 2021; Bellot‐Saez et al. 2017; Verkhratsky et al. 2023). Moreover, there is a growing appreciation that astrocytes are directly involved in the modulation of neuronal activity and information transfer. For example, uncaging calcium in hippocampal and layer V cortical astrocytes induces the broadening of action potentials, regulation of axon initial segment (AIS) excitability, and modulation of conduction velocity in the axons they contact (Lezmy et al. 2021; Sasaki et al. 1979). In the retina, mechanical stimulation elevates astrocytic calcium and affects the excitability of ganglion cells contacted by those astrocytes (Newman and Zahs 1998).

Because mechanical stimulation of the retina induces astrocyte‐mediated modulation of neural excitability, it is likely that a similar phenomenon can be induced through more physiologically relevant stimuli. This would have consequences for neural encoding and the transfer of visual information from the retina to the CNS projection targets of the optic nerve. Critically, the structure and placement of elements of the astrocyte microstructure appear amenable to modulating ganglion cell excitability or monitoring their electrical activity, as explored in our recent paper (Holden et al. 2023). It is possible that electrical activity in astrocyte microstructural motifs drives the modulation of ganglion cell physiology observed through mechanical stimulation. The motivation for our current study is to better understand naïve retinal astrocyte electrophysiology in mice at the whole‐cell level so we can eventually understand regional nuances in any electrical responses of their microstructural motifs.

Clark and Mobbs reported voltage clamp recordings of astrocytes in rabbit retina and found their current–voltage (IV) relationships show high permeability to potassium and sodium at the whole‐cell level (Clark 1993; Clark and Mobbs 1994). These relationships are similar to those observed in Müller glia of various species including mouse (Newman 1985a; Reichelt et al. 1993; Pannicke et al. 2017). Moreover, current clamp recordings show that Müller cells have a response to light caused by the uptake of potassium from the plexiform layers (Conner et al. 1985; Witkovsky et al. 1985). There, light stimulation increases neuronal activity, releasing potassium which is rapidly cleared by Müller cells and siphoned to the vitreal surface (Karwoski et al. 1989; Kline et al. 1985; Dick et al. 1985; Newman et al. 1979). This process evokes an electrical response with depolarizations to light onset and offset which track extracellular potassium levels. Müller cells additionally depolarize to light through elevations in intracellular calcium (Newman 2005; Rillich et al. 2009). This likely occurs in response to ATP signaling, but there is some evidence for intrinsic photosensitivity in some species (Newman 2005; Marchese et al. 2022). We might expect astrocyte responses to be similar given their role in potassium buffering throughout the CNS and their sensitivity to extracellular ATP (Bellot‐Saez et al. 2017; Newman 2001; Wallraff et al. 2006; Murakami and Kurachi 2016). If retinal astrocytes depolarize in response to light, it may initiate processes to regulate neuronal excitability.

Here, we conduct patch clamp single unit recordings from astrocytes and Müller glia in the intact mouse retina to investigate responses to light and voltage steps at a whole‐cell level. We include Müller cells in our recordings because they have similar transcriptomic profiles as astrocytes and perform functions characteristic of astrocytes in other regions of the CNS, including their direct contact with neurons at synapses in the plexiform layers where astrocytes are not present (Cullen et al. 2024; Lukowski et al. 2019; Tworig and Feller 2022). We compare responses to determine if both classes of cells have conserved electrophysiology in the retina. We find that astrocytes depolarize to light stimulus independently of neuronal action potentials with similar waveforms to Müller glia endfeet. This depolarization is due to potassium uptake, likely released by Müller cells, and is not a result of internal calcium release. Furthermore, both astrocytes and Müller cells exhibit rectifying IV relationships in response to voltage step, and the polarity of rectification can change with repeated stimulation.

## 2 Methods

### 2.1 Patch Clamp Electrophysiology

Mice were euthanized by cervical dislocation and eyes rapidly enucleated under red light conditions as described previously (630 nm, 800 μW/cm^2^, FND/FG, Ushio) (Holden et al. 2023, 2024). Retinas were dissected in a carbogen‐saturated Ames' medium (US Biologic, A1372‐25) supplemented with 20 mM D‐glucose and 22.6 mM NaHCO_3_ (pH 7.4, 294 Osm). The potassium concentration of the Ames' media is 3.6 mM. During recordings, retinas were perfused by Ames' media at a flow rate of 3 mL/min at 30°C (Warner Instruments, TC‐344C). Individual tdTomato‐positive astrocytes and Müller endfeet were identified using a 40X water immersion objective on an Olympus BX50 microscope. Cells were patched in a whole‐cell configuration using a borosilicate pipette (I.D. 0.86 mm, O.D. 1.5 mm; Sutter Instruments) filled with (in mM) 125 K‐gluconate, 10 KCl, 10 HEPES, 10 EGTA, 4 Mg‐ATP, 1 Na‐GTP, 0.1 Alexa 488, or 0.8 Lucifer Yellow dye (Invitrogen L453, A10436). The intracellular solution pH was 7.35 and osmotic concentration was between 285 and 295 Osm. Cells were patched in voltage clamp mode to aid in the formation of a successful GΩ seal. Astrocytes and Müller glia were initially held at −80 mV and RGCs at −65 mV.

Recordings were made using Clampex 10.7, Multiclamp 700B, and Nis Elements Br 4.20 software. Light stimulation recordings were performed in current clamp mode and stimulated with a 525‐nm light. Light stimulation was mediated through a pE‐4000 from Andover (United Kingdom). The light was applied as a full‐field stimulus for 3 s at 3.4 mW/cm^2^. Raw data were sampled at 10,000 Hz and analyzed using the Python library PyABF and smoothed using a Savitzky–Golay filter with window length 300 and polynomial order 3. IV relationships were determined by recording cells held at −80 mV while stepping to voltages ranging from −95 to +35 mV in steps of 10 mV for 50 ms. Current values were taken as the average current observed within the middle 40 ms of the 50‐ms stimulation period. Raw data were sampled at 50,000 Hz. Potassium recordings were performed in current clamp mode while spraying a solution of Ames' media supplemented with an additional 1.4‐mM solution of KCl (5.0 mM total) using a Picospritzer II. BaCl_2_ recordings were made by direct application of a solution of BaCl_2_ in Ames' media into the recording chamber at a final concentration of 1 mM. Recordings with 4,9‐anhydro‐tetrodotoxin (aTTX) were performed by bath application in Ames' media to a final concentration of 500 nM (Tocris, #6159). Bath addition and incubation occurred in the absence of media flow. Wash out was initiated with the return of media flow through the recording chamber. Gap junction blockade recordings were performed by transfer of the inlet media line from normal Ames' media to a solution of the inhibitor dissolved in Ames' media. The solution of meclofenamate was prepared by dissolving 25 mg of meclofenamic acid sodium (Thermo Fisher Scientific #J60484.06) into 250‐μL DMSO and then adding this dropwise into Ames' media to a final concentration of 200 μM. Carbenoxolone solution was prepared directly into Ames' media to a final concentration of 200 μM (Millipore #C4790). Octanol solution was prepared directly into Ames' media to a final concentration of 500 μM (Thermo Fisher #A15977.AP).

Firing rates for ganglion cells were determined using the *signal.find_peaks* function from the Python library Scipy, looking for spikes with a prominence of at least 20 mV.

### 2.2 Calcium Imaging

Vitreous was removed from each retina as described above, and the tissue was incubated in a solution of Fluo‐4 AM (31 μg/mL) and pluronic F127 (2.6 mg/mL, Biotium #59004) in Ames' media for 30 min in a carbogen‐saturated container. Retinas were then transferred to a recording bath and imaged with excitation of 490 nm. The imaging light served as the excitation source. After 5–6 s of image acquisition, a solution of ATP (0.1 mM) was puffed into the recording frame, and recording continued for another ~25 s. Because many cells (both astrocytes and Müller cells) exhibit calcium responses to ATP, frames following its application serve to identify cell types. Regions with calcium fluorescence were outlined in ImageJ, and the intensity profiles within those regions of interest over time were determined with a Python script.

### 2.3 Potassium Diffusion Modeling

Potassium diffusion was assessed using Fick's second law in two dimensions $r^{2}=\frac{4D*t}{\lambda^{2}}$ where r is the distance diffused in time t. The diffusion coefficient D for K^+^ was taken as 1.96 μm^2^/ms (Chung et al. 1999). For free diffusion, *λ* = 1 and for restricted diffusion, *λ* was taken as 1.55 (Odette and Newman 1988). Modeling was simplified using two dimensions instead of 3.

To determine the expected voltage change in an astrocyte in response to neuronal activity, we modeled two conditions‐ one for an astrocyte contacting few axons and another for astrocytes covering axon bundles. In the first case, we assumed the RGC axonal efficiency was comparable to unmyelinated hippocampal Mossy fibers (1.6 pmol Na^+^/ cm^2^ per action potential) and that the quantity transferred of Na^+^ and K^+^ were similar (Alle et al. 1979). We assumed an average axon diameter of 0.8 μm, a firing frequency of 100 Hz, and a 1:1 ratio of ON to OFF RGCs based off the literature and our own observations (Boal et al. 2022). The receptive region of potassium diffusion was determined by our latency measurements and Fick's law. However, because the total volume of the diffusion compartment was unknown, we plotted voltage change as a function of both axon number and depth of the diffusion compartment. Because the tracing of axons through the receptive region is irregular, we modeled the length of each axon through it as the average geometric chord length. Total potassium entering the diffusion compartment volume after 1 s of stimulation was input to the Goldman–Hodgkin–Katz (GHK) equation along with the concentrations of ions in the intracellular recording solution and Ames' media to determine voltage change. An identical approach was taken for modeling voltage change for astrocytes on RGC axon bundles, but we corrected for the fact that not all the potassium would diffuse out of the bundle to reach the astrocyte. To determine what fraction of K^+^ makes it to the surface, we created a simulation of potassium release from six points along each axon in an 18 × 18 grid (bundle). We chose an 18 × 18 bundle based off our own counts of axon bundles coalescing at the optic nerve head and total RGC counts per retina. We simulated random walks for each point of release to determine what fraction of points ‘walks’ into the receptive compartment within 1 s. Taking the average fraction reaching the surface for 10‐ms time bins (100‐Hz firing rate) gives us the fraction of total potassium released which could reach the bundle surface and influence astrocyte voltage (~78%).

### 2.4 Curve Fitting

IV relationships were fit using a linear combination of curves (type‐A potassium, calcium, and calcium‐activated potassium currents) from dissociated *Ambystoma tigrinum* Müller glia reconstructed from (Newman 1985a). Each of the three curves from the Newman 1985 paper was multiplied by a weight which was set to vary between 0 and 1. One million runs of setting each weight randomly were conducted, and minimization of the sum of square error between our experimental data and the data from the Newman paper determined the final fit parameters.

### 2.5 Cell Density

RGC cell locations were determined from an RBPMS‐labeled mouse retina using RGCode (Masin et al. 2021). The distance of each cell to the optic nerve head was then determined. For each cell, density values were determined as the number of cells within a 100‐μm radius (extrapolated to a 1‐mm^2^ area). Cells were not included in the analysis if they were less than 100 μm from the tissue edge. Linear axon density was determined based on the RGC soma distribution. Because all RGCs send an axon to the optic nerve head, at each eccentricity we have a measurement of how many RGCs are beyond that eccentricity and, by extension, the number of axons which must pass by. Linear axon density was determined for rings centered at the optic nerve head as the number of RGCs that have a greater eccentricity than the ring border, divided by the path length of that ring which is within the bounds of the retina. This measurement is an averaged value that does not account for axon fasciculation.

Müller glia cell density was determined using Sox9 labeling in the inner nuclear layer of a wholemount retina (Wang et al. 2017). Images were enhanced for contrast in ImageJ and cell locations were found using the “*Find Maxima*” function. Density estimates were then found in the same way as RGC soma.

### 2.6 Statistics

All data were tested for normality using the D'Agostino and Pearson test. Failing normality conditions, significant differences were determined using either a Mann–Whitney test or Kruskal–Wallis test followed by Dunn's multiple comparisons depending on group size. If normally distributed, group differences were determined using a *t*‐test or one‐way ANOVA followed by Tukey's post hoc test. Statistical tests were conducted in GraphPad Prism 10.2.2.

## 3 Results

### 3.1 Astrocytes Demonstrate Small Amplitude Graded Depolarization to Light ON and OFF

**FIGURE 1:**
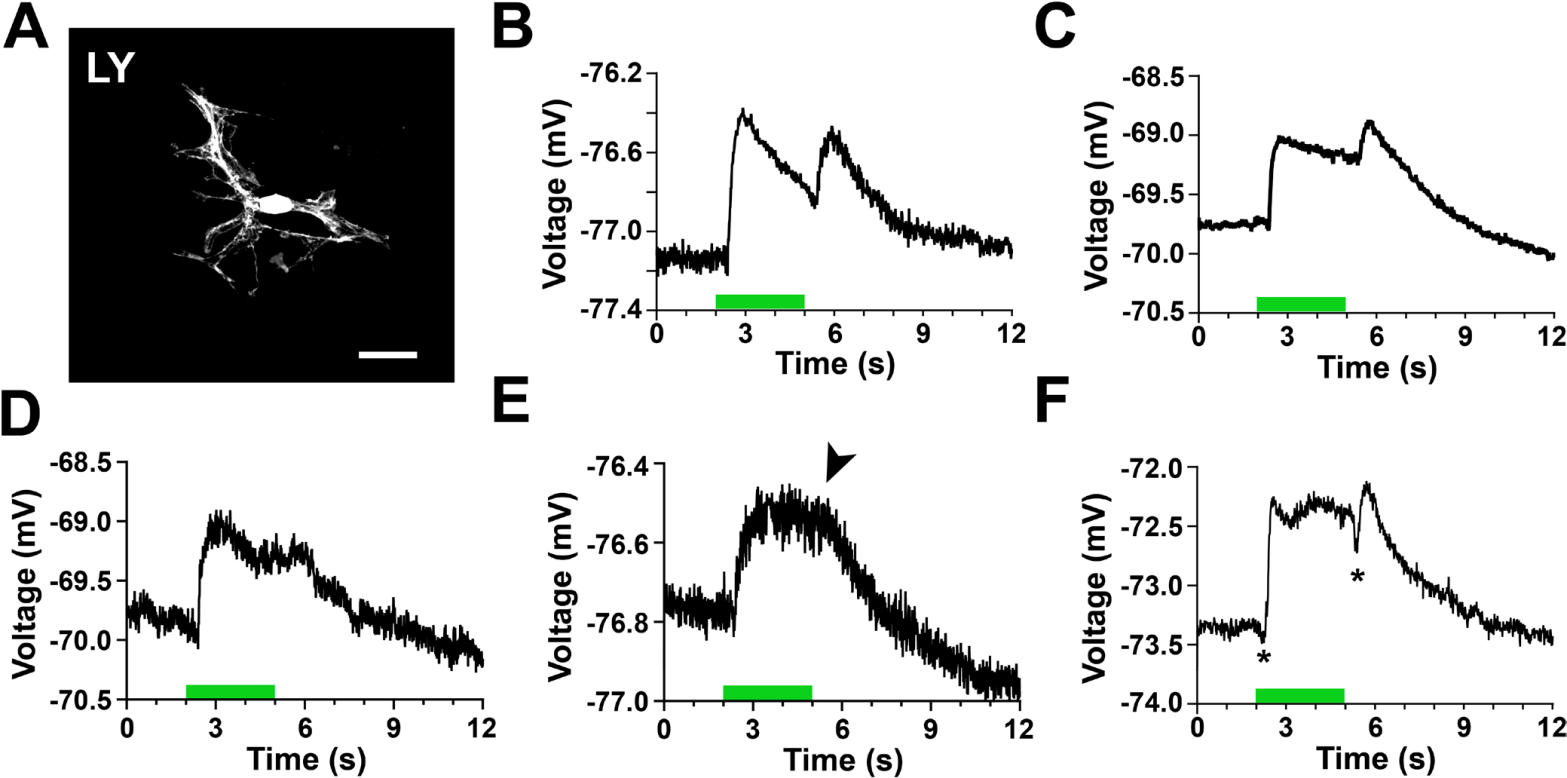
Astrocytes demonstrate small amplitude, graded depolarization to light stimulus. (A) Example astrocyte recorded from in current clamp mode and filled with lucifer yellow dye, scale 25 μm. (B–D) Astrocytes depolarize to light onset and offset with variability in voltage stability during light presentation. A 3‐s 525‐nm light stimulus is indicated by the green bar. (E) An example astrocyte trace lacking a distinct response to light offset (black arrow). (F) An example astrocyte trace where brief hyperpolarization precedes depolarization to both ON and OFF stimuli. *marks hyperpolarization.

We targeted astrocytes in the nerve fiber layer (NFL) for whole‐cell voltage recordings and intracellular dye filling using a transgenic mouse line expressing tdTomato under the control of the glial fibrillary acidic protein (GFAP) promoter, as described in our recent paper (Holden et al. 2024). All astrocytes depolarize to light onset and most (86%) to light offset, with variability in voltage stability between both stimuli Figure 1A–E. Rarely, an astrocyte briefly hyperpolarized to light onset and offset prior to depolarizing to each stimulus (3% cells; Figure 1F).

### 3.2 Astrocyte Light Response Is Dependent on Extracellular K^+^ but Not Voltage‐Gated Sodium Channels

**FIGURE 2:**
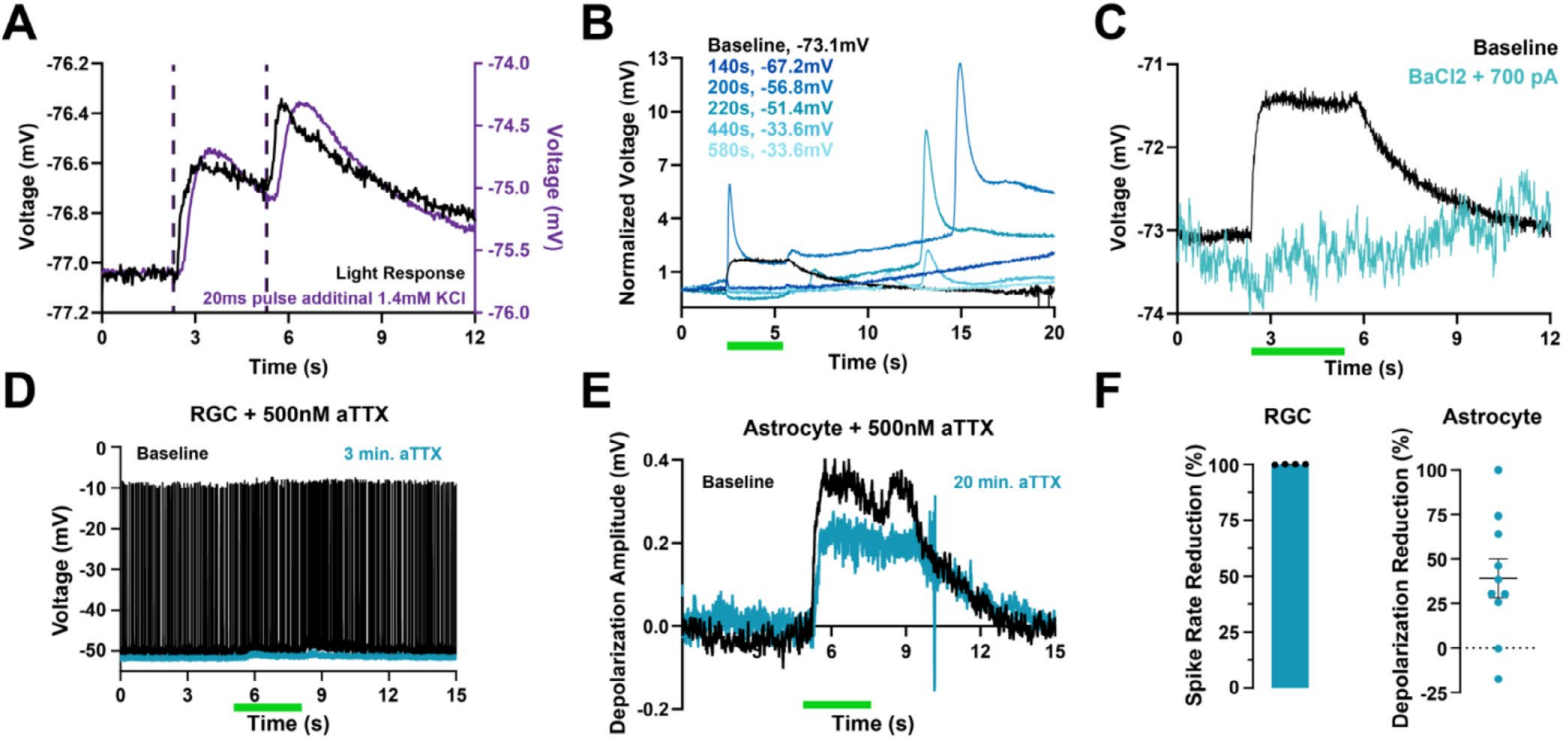
Astrocyte light response is dependent on extracellular K+ but not voltage‐gated sodium channels. (A) An astrocyte response to 525‐nm light is plotted in conjunction with the voltage change from picospritzing an Ames' solution with an additional supplemental 1.4 mM of KCl for 20 ms from 112 μm near the same cell. Dashed lines show the onset and offset of light and Picospritz pulses. (B) Light response is disrupted and eventually abolished when potassium channels are blocked by 1 mM of barium chloride. Each curve is a light response trace from the same astrocyte at a specific timepoint following bath application of BaCl_2_. In the legend, the time at which the recording begins is noted along with the starting voltage for that trace. Each plotted trace is baseline corrected to 0 mV starting voltage, as BaCl_2_ depolarizes the cell. (C) Injection of current to bring the cell back to its pre‐BaCl_2_ RMP does not restore the light response. Traces in B and C are from the same astrocyte. Three cells from different mice were recorded. (D–F) 500 nM of tetrodotoxin rapidly abolishes retinal ganglion cell spiking (< 5 min, 99.95% ± 0.05% spike reduction), but at a 20‐min incubation, most astrocytes retain a light response (9/10 cells, ON response amplitude % of baseline: 60.8% ± 10.9%, AVG ± SEM).

The waveform of the astrocyte light response is similar in shape to light‐induced potassium increases in the plexiform layers of the retina, which are buffered by Müller glia. We wanted to determine whether potassium release during neuronal activity in the NFL is similarly responsible for astrocyte depolarization. In Figure 2A, we applied local KCl using a Picospritzer and were able to not only induce depolarization but also replicate the overall waveform of the astrocyte light response curve by pulsing the potassium solution twice. Pulse duration correlated well with depolarization magnitude (*R* ^2^ = 0.9884). Additionally, blocking potassium channels with BaCl_2_ disrupts and ultimately abolishes the light response Figure 2B. Because BaCl_2_ depolarizes astrocyte RMP, we wanted to verify that the lack of light response was not simply due to a lack of driving force for potassium to enter the cell. We injected current into BaCl_2_‐depolarized astrocytes to restore their baseline RMP levels, and light responses were not rescued Figure 2C.

Because action potentials are a significant source of potassium in the NFL (Newman 1985b), we blocked voltage‐gated sodium channels using anhydro‐tetrodotoxin (aTTX, 500 nM) to test whether the astrocyte light response would be abolished. As expected, even brief (5 min) application of aTTX prevented ganglion cells from firing action potentials (99.95% ± 0.05% reduction, Figure 2D). Surprisingly, however, astrocytes on average retained 60.8% ± 10.9% of their original depolarization amplitude in response to light, even after a far longer (20 min) exposure to aTTX (Figure 2E,F). In contrast to ganglion cells, this 39% decrease corresponded to only a fraction of a millivolt in amplitude.

### 3.3 The Astrocyte Light Response Resembles That of Müller Glia

**FIGURE 3:**
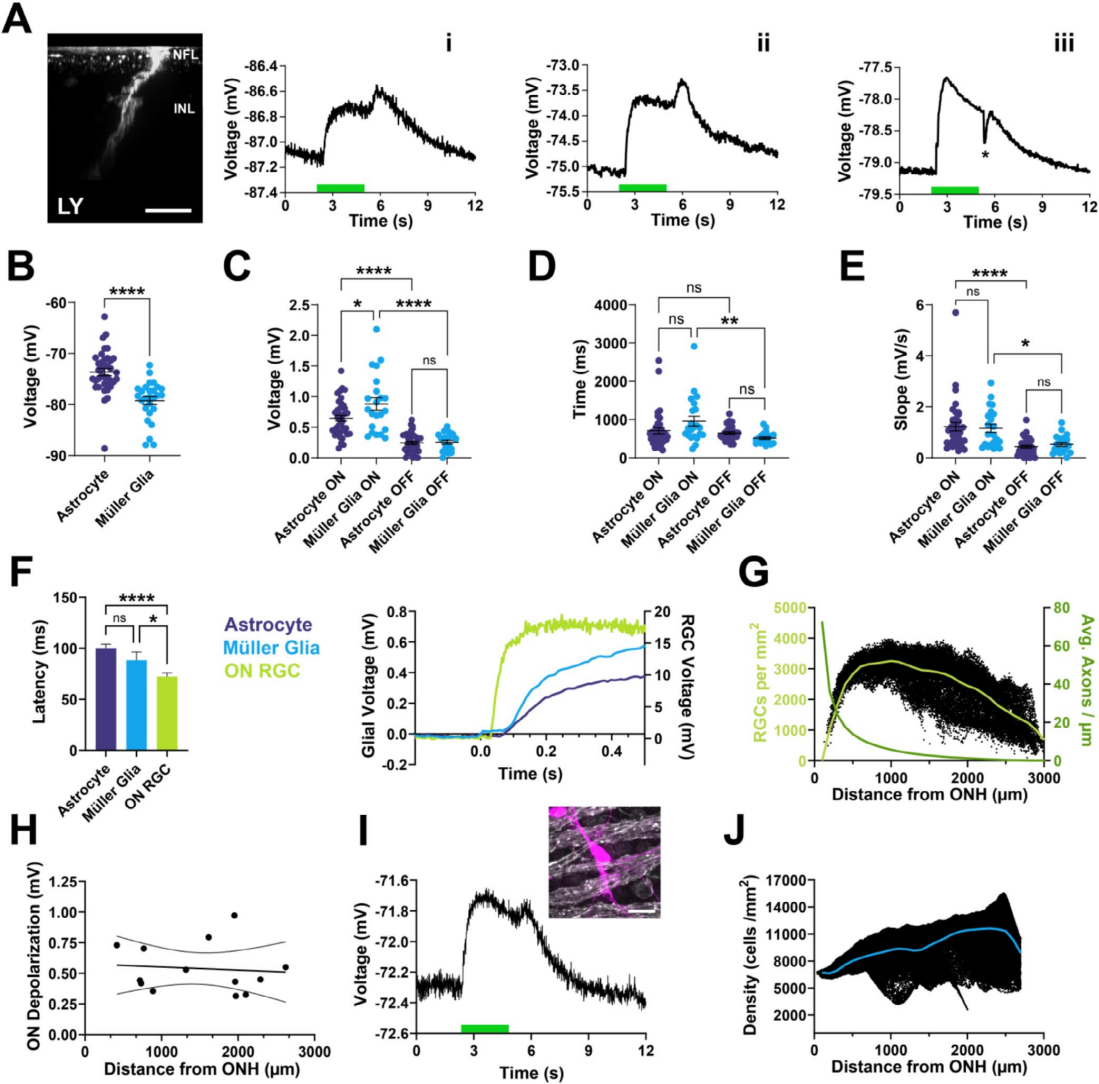
The astrocyte light response resembles that of Müller glia. (A) Three representative Müller glia endfoot current clamp recordings in response to 525‐nm light. Cell identity was verified through dye filling. Scale is 25 μm. (B–E) The kinetics of the astrocyte and Müller glia light responses are similar. (B) Müller glia and astrocytes differ in RMP (*p* < 0.0001, Mann–Whitney). (C) The magnitude of depolarization differed both between cell types (A‐ON vs. MG‐ON, *p* = 0.0131) and also between light onset and offset within a given cell type (A‐ON vs. A‐OFF *p* < 0.0001, MG‐ON vs. MG‐OFF *p* < 0.0001). 1‐way ANOVA + Tukey post hoc. (D) Time to reach depolarization peak was only different between MG ON and OFF responses (*p* = 0.0023, Kruskal–Wallis + Dunn's multiple comparisons). (E) Depolarization rate was not significantly different between groups. However, both macroglia have a slower depolarization rate to light offset than onset (astrocytes *p* < 0.0001; MG *p* = 0.0136). Kruskal–Wallis + Dunn's multiple comparisons. (F) Averaged light response curves for α‐ON RGCs, Müller glia, and astrocytes with semi‐manual determination of response latency (glial voltage signal increases 1.5 standard deviations above baseline; peak location for first RGC action potential). Response latencies for ON RGCs, Müller glia, and astrocytes were 72.3 ± 3.5, 88.4 ± 8.1, and 100.0 ± 4.1 ms, respectively (trace count: RGC 124, Müller glia 25, astrocyte 63. *p* < 0.0001 A‐RGC; *p* = 0.0170 MG‐RGC, Kruskal–Wallis + Dunn's multiple comparisons). Bars are mean ± SEM. (G) Area density of RGC soma and linear density at eccentric rings for axons crossing on a path to the ONH. (H) Amplitude of astrocyte depolarization to light is not dependent on eccentricity (*n* = 13). (I) Example patched astrocyte near multiple RGC axon bundles which has a light‐induced depolarization amplitude of 0.62 mV. Magenta (Lucifer yellow) shows an astrocyte, and white shows axons (beta iii tubulin). Scale is 20 μm. (J) Müller glia density as a function of eccentricity is relatively constant.

Through the GFAP promoter, tdTomato also labeled a few Müller glial cells, allowing us to record from their endfeet in the NFL (Figure 3Ai–iii). As with astrocytes, all Müller cells depolarized to light onset and most to light offset (91%), with similar response profiles and kinetics as astrocytes Figure 3A–E. Endfeet sometimes hyperpolarized to light offset prior to larger amplitude depolarization (4% cells, Figure 3Aiii). Both astrocytes and Müller glia are hyperpolarized in the dark, with an average resting membrane potential (RMP) of −73.6 and −79.2 mV, respectively (Figure 3B). Greater differences in response kinetics were observed to ON and OFF stimuli than were between cell types; however, the Müller ON response amplitude was greater than that of astrocytes (37% larger, *p* = 0.0131, Figure 3C). For both cell types, depolarization to light offset was significantly smaller than depolarization to onset (*p* < 0.0001, Figure 3C), while the time to peak was largely the same (Figure 3D). This is reflected in the greater rate of depolarization at onset than offset for both astrocytes and Müller glia (*p* ≤ 0.0164, Figure 3E), neither of which differed between cell types (ON *p* = 0.9883; OFF *p* = 0.9673). Astrocytes and Müller glia additionally share similarities in their time to depolarization onset (ON latency). Astrocyte ON latency (100.0 ± 4.1 ms) is more similar to that of Müller glia (88.4 ± 8.1 ms, *p* = 0.9999) than to that of ON RGCs (72.3 ± 3.5 ms, Figure 3F; *p* < 0.0001).

### 3.4 Astrocyte Depolarization Amplitude Is Not Dependent on Retinal Eccentricity

Whole‐cell dye filling allowed for the recovery of cell location following recordings. While the density of RGC axons increases exponentially approaching the optic nerve head (Figure 3G), the amplitude of astrocyte depolarization does not vary with retinal eccentricity (*R* ^2^ = 0.008; Figure 3H,I) as would be expected if a significant portion of the response were due to RGC‐derived potassium. Even astrocytes patched directly on axon bundles near the optic nerve head were unremarkable in their depolarization amplitude Figure 3I. In contrast, the sampling density of Müller glia changes little with retinal eccentricity, despite a high level of variability (Figure 3J) (Wang et al. 2017). The invariance in the astrocyte depolarization amplitude and Müller cell density with eccentricity, along with their similar response profiles, compelled us to consider a potential link between astrocyte depolarization and potassium release from Müller cells.

### 3.5 Astrocytes and Müller Glia Have Common Current–Voltage Rectification Patterns That Change With Repeated Stimulation

**FIGURE 4:**
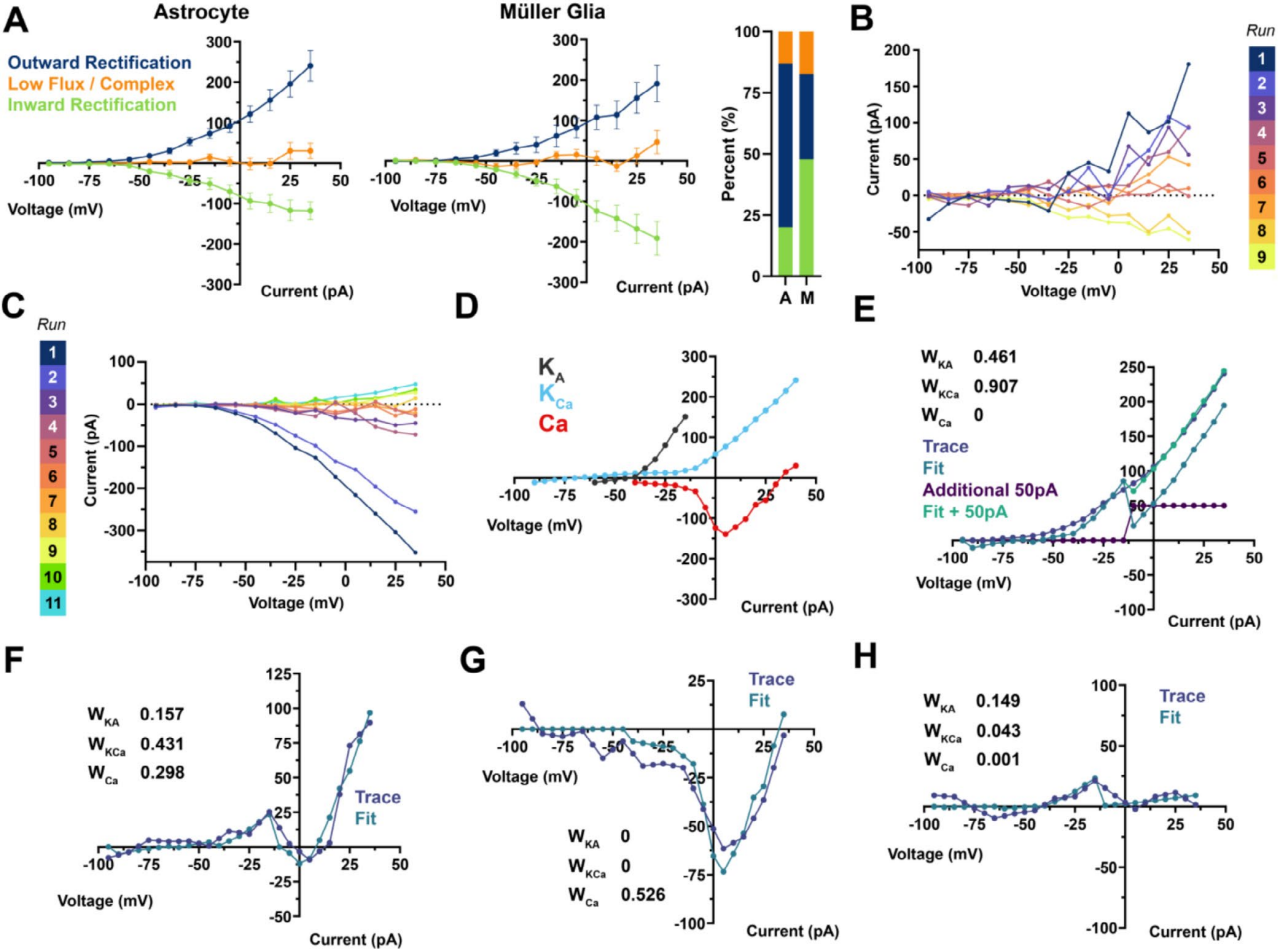
Astrocytes and Müller glia share diverse rectification patterns which adapt to repeated stimulation. (A) Astrocytes and Müller glia can be found in varying states of ionic permeability. Whole‐cell voltage clamp recordings in response to voltage step from a holding potential of −80 mV show current–voltage relationships exhibiting outward, low, and inward rectification polarity (Number of astrocytes: Outward 30, low flux 6, inward 9. Number of Müller glia endfeet: Outward 8, low flux 4, inward 11). Points indicate mean and bars indicate SEM. Both cell types have similar linearly interpolated reversal potentials for both outward and inward current (astrocyte: Outward −71.2 ± 0.6 mV, inward −65.2 ± 1.6 mV. Müller glia: Outward −69.6 ± 0.8 mV, inward −67.9 ± 0.7 mV). Astrocytes (B) and Müller glia (C) can switch between states of ionic permeability in a stimulus‐dependent manner. Repeated acquisition of the voltage step protocol changes the current–voltage relationship. Astrocyte rectification curves are well fit by potassium and calcium currents observed in salamander Müller glia. (D) Re‐created data from Newman 1985 showing isolated potassium and calcium currents from enzymatically dissociated salamander Müller glia. (E–H) Least‐squares regression between weighted curves in D and example individual astrocyte IV curves. Traces in E–H are outward rectification, shifted outward rectification, complex, and low flux respectively. Many traces can be well approximated. Inward rectification curves in A, B could not be well approximated due to a lack of inward current in the positive voltage region of the Newman curves. *R* ^2^ values for outward fit, outward fit +50 pA correction, shifted outward fit, complex fit, and low flux fit are as follows: 0.71, 0.99, 0.92, 0.85, 0.06 (noise @ ~0pA).

To investigate the similarity in astrocyte and Müller glia electrophysiology further, we recorded current flux in response to voltage steps in both cell types. Most cells (87% astrocytes and 83% Müller cells) displayed current rectification Figure 4A. Both outward and inward rectification were observed for both cell types, and the overall shape of the rectification patterns was similar Figure 4A. Linearly interpolated reversal potentials for both outward and inward current were similar between cell types (astrocyte: outward −71.2 ± 0.6 mV, inward −65.2 ± 1.6 mV. Müller glia: outward −69.6 ± 0.8 mV, inward −67.9 ± 0.7 mV). In all cells, from −95 mV to the reversal potential, negligible current flowed. At the reversal potential, the relationship for most cells is linearly sloping in either the positive or negative direction. However, a subset of cells was not responsive and passed low current at all potentials. Interestingly, when the voltage step protocol was repeated in close succession, the rectification pattern could be made to decay to zero and eventually flip polarity Figure 4B,C. Individual astrocyte complex and outwardly rectifying curves are well fit by potassium and calcium currents observed in dissociated salamander Müller glia, demonstrating conserved electrophysiology across species and cell types while also supporting a potassium origin for the astrocyte light response Figure 4D–H (Newman 1985a). However, the inward rectification plot could not be well‐fit by these curves (not shown). Additionally, some recorded IV curves were more complex than the averages shown in Figure 4A. These more complex fits were rare (17.5%; Figure 4F–H).

### 3.6 Astrocyte Light Response Is Not due to Calcium

**FIGURE 5:**
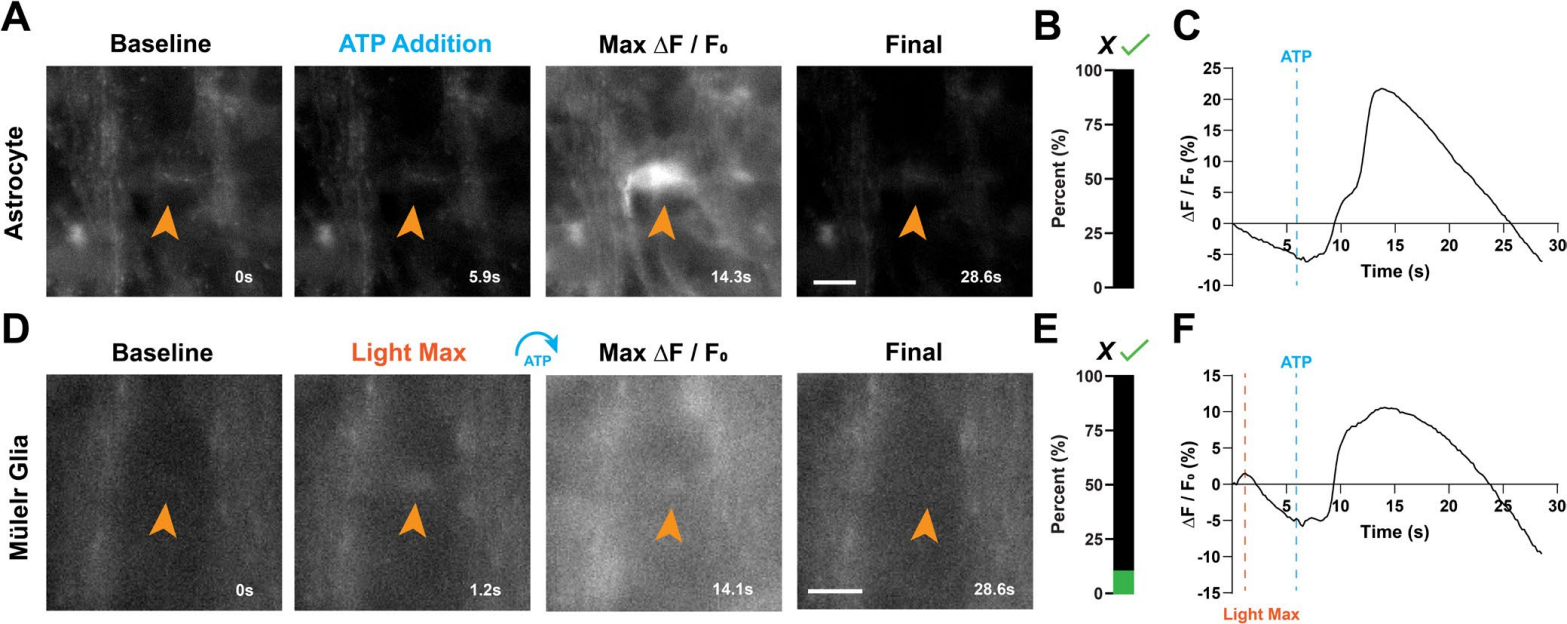
Astrocyte light response is not due to calcium. (A–C) Astrocytes show an increase in intracellular calcium in response to ATP but not light. Example fluorescence time course in A. Scale 10 μm. (B) 0/170 astrocytes in 73 total frames of view (frame area: 224 × 168 μm^2^, 3 mice/4 retinae) depolarized to light. (C) Fluorescence profile of representative astrocyte. (D) Müller glia endfeet show calcium response to both light and ATP. (E) A light response was observed in Müller cells in 8/73 total frames of view (224 × 168 μm^2^, 3 mice/4 retinae). Scale 5 μm. (F) Fluorescence profile of representative Müller cell. For each cell, fluorescence intensity was determined for pixels in an ROI within the cell body or endfoot, respectively. 490‐nm light served as both the excitation and recording light, and it was verified that astrocytes and Müller glia depolarize to 490‐nm light as they do for 525 nm (not shown). In A and D, the second frame differs for the example astrocyte and Muller endfoot. Because the astrocytes lack a light response, we show the point of ATP addition (Noted by cyan line in C). For the Muller endfoot, we show a frame “Light Max” demonstrating the light response which is more informative than the timepoint where ATP is released (simply a darker frame). For both cells, the Max ΔF/F_0_ is the maximum of the time course after ATP addition.

Because of previous reports that Müller glia exhibit light‐induced calcium depolarizations in rats, we wanted to verify that the astrocyte light response is not due to calcium (Newman 2005). While incubating ex vivo retinas with Fluo‐4 AM, we observed light‐induced calcium increases in mouse Müller cells but did not see evidence for this in astrocytes Figure 5. Bath and Picospritz application of ATP caused depolarization and robust increases in calcium fluorescence in both cell types. Because we observe fluorescence increases in Müller cells in response to light and not astrocytes, but the magnitude of depolarization for each cell in response to light is similar, we would expect to see an increase in fluorescence in astrocytes if the light response were due to calcium.

### 3.7 Potassium Diffusion Modeling Supports a Dual Origin of the Astrocyte Light Response

Possible sources of potassium for an astrocyte light response are neuronal or Müller glial in origin. Regardless of the source, the potassium would need to diffuse from that source to the astrocytes to influence them electrically. Here we use the difference in latency (Δlatency) from light onset to depolarization between each pair of cell types (astrocyte–ganglion cell and astrocyte–Müller cell) along with an estimate for the rate of potassium diffusion through the NFL to define a distance within which Müller and ganglion cells could influence the astrocyte potential through a diffusion‐mediated potassium mechanism. We used this distance along with a model of RGC potassium release during action potential firing to see if either a neuronal or Müller origin of potassium was consistent with our recordings.

**FIGURE 6:**
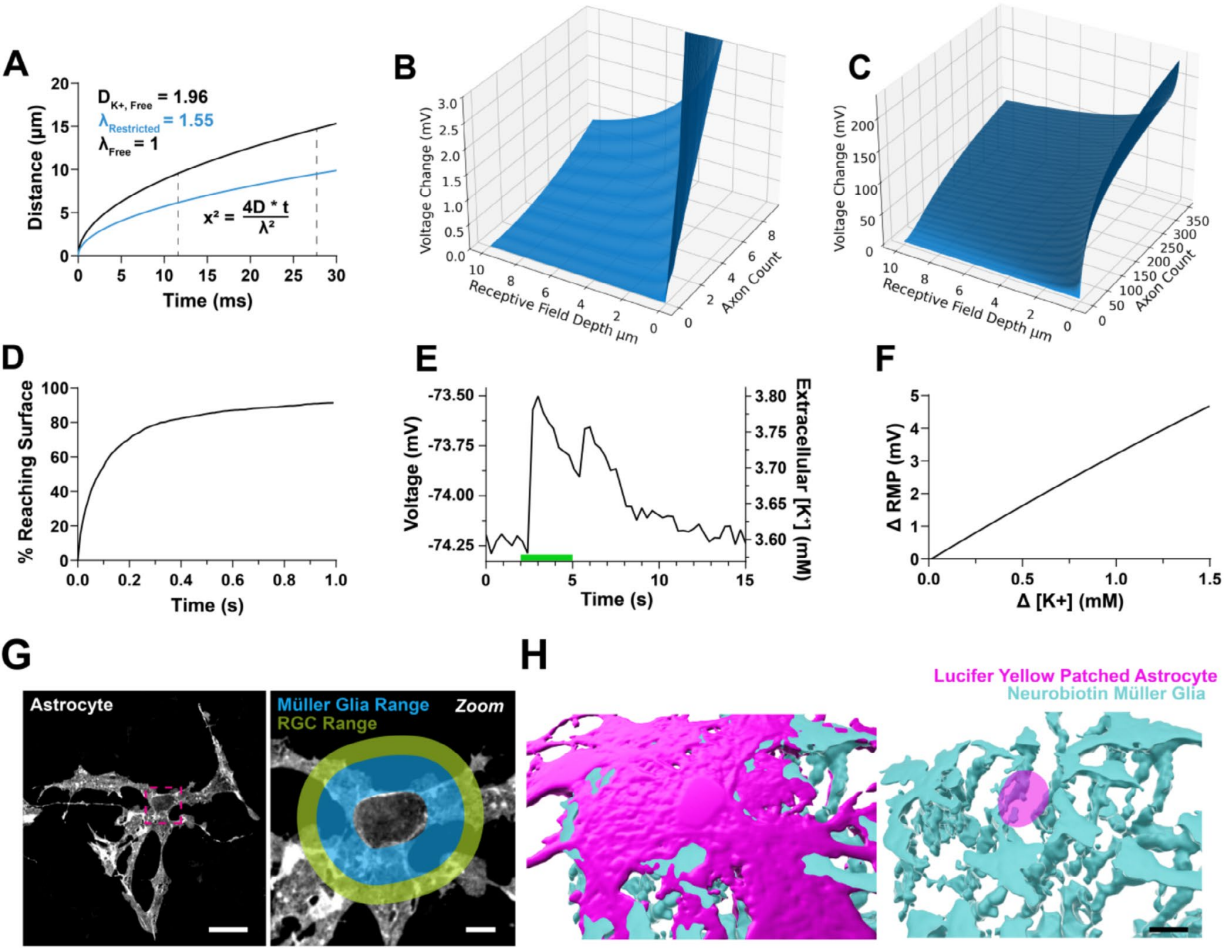
Potassium diffusion modeling supports a dual origin of the astrocyte light response. (A) Estimate of free and restricted diffusion of potassium using Fick's law. Using the Δlatency in light‐induced depolarization for astrocyte‐RGC (27.7 ms) and astrocyte‐Müller glia (11.6 ms), the free diffusion distance of potassium in this time is 14.7 and 9.5 μm, respectively. Restricted diffusion distances are 9.5 and 6.2 μm. (B, C) Simulated change in astrocyte voltage as a function of the number of axons within a distance bounded by restricted potassium diffusion from RGCs. The potassium released per unit surface area of an axon was estimated from the literature and voltage change estimated using the Goldman–Hodgkin–Katz (GHK) equation. (D) Random‐walk simulation of potassium which reaches the surface over time for an 18 × 18 bundle of axons. (E) An astrocyte voltage response to 525‐nm light is shown with an estimate of how the extracellular concentration of K+ would have to change to fit that curve (GHK equation). (F) Plot of how RMP changes with changing extracellular potassium (GHK model). (G) Confocal image of an astrocyte illustrating the restricted diffusion distance of potassium for RGC and Müller cell release. The green region shows the distance from the edge of the cell body that RGC‐originating potassium could diffuse to reach the edge of the astrocyte cell body within the Δlatency period. The cyan region illustrates this distance for Müller‐derived potassium. Scale for full size image is 20 μm and for the zoomed image is 5 μm. (H) Patched astrocyte with gap junction‐mediated spread of dye to Müller glia. Lucifer yellow primarily labeled the astrocyte (magenta) while neurobiotin primarily labeled the Müller glia. Circled region indicates the astrocyte cell body. Scale is 10 μm.

We looked at depolarization latencies for astrocytes, Müller endfeet, and α‐ON retinal ganglion cells (RGCs) in Figure 3F. Approximating the NFL system as two‐dimensional and applying Fick's second law $r^{2}=\frac{4D*t}{\lambda^{2}}$ allowed us to determine the maximum distance of potassium diffusion in each Δlatency time period Figure 6A. Using the Δlatency for astrocyte‐RGC (27.7 ms) and astrocyte–Müller glia (11.6 ms), the restricted diffusion distances for RGC and Müller‐derived potassium are 9.5 and 6.2 μm, respectively (Figure 6A) (Odette and Newman 1988).

We modeled potassium release from RGC axons by assuming their axonal efficiency is similar to other unmyelinated axons in the CNS and estimating their firing rate and geometric properties from our own observations and those reported in the literature (Odette and Newman 1988; Alle et al. 1979; Boal et al. 2022). Using restricted diffusion times and the GHK equation, we modeled astrocyte voltage change as a function of the number of axons passing within an astrocyte's immediate vicinity. For a small number of axons, this estimate of voltage change falls within the range of what we observe experimentally Figure 6B. However, when modeling astrocytes on axon bundles, the estimates for voltage change far exceed this limit Figure 6C,D.

We additionally used the GHK equation to determine the change in extracellular potassium which would be needed to induce the light response Figure 6E,F. On average, the elevation in extracellular potassium would need to be 0.19±0.01 mM to recreate the astrocyte ON response. This is similar in magnitude to measurements of potassium efflux from dissociated Müller glia endfeet (~0.1 mM) following stimulation of the distal portion of the cell with KCl (Newman et al. 1979). We then used measurements of average astrocyte soma size (83.3 ± 0.8 μm^2^, *n* = 1983 cells) and Müller glia density (10,145 ± 5 cells/mm^2^, *n* = 174,021) to estimate that on average 4.6 Müller cells fall within the Müller‐astrocyte Δlatency‐defined diffusion area (6.2‐μm radius). It follows then that the concentration of extracellular potassium would elevate between 0.1 and 0.46 mM, which is consistent with concentrations needed to replicate the astrocyte light response. These measurements also estimate that 84% of astrocytes have cell bodies contacted by Müller endfeet Figure 6G,H. This is supported by our observation that a significant proportion of our attempts to patch astrocytes instead patch Müller glia endfeet which wrap the astrocyte cell body (36%).

**FIGURE 7:**
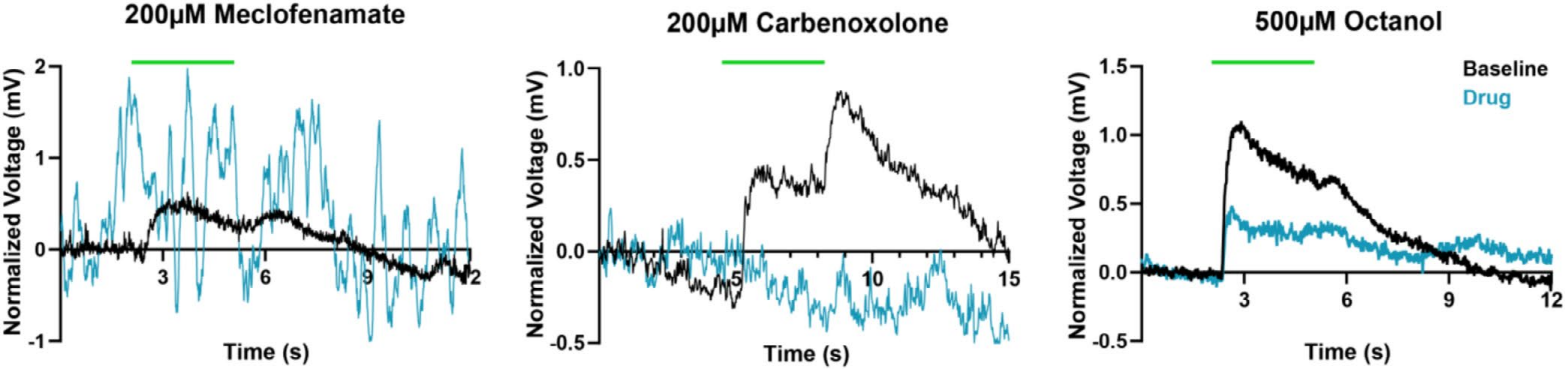
Astrocyte light response is sensitive to gap junction blockade. 200 μM of meclofenamate, 200 μM of carbenoxolone, and 500 μM of octanol all disrupt the astrocyte light response. Plots show current clamp whole‐cell recordings of astrocytes in response to 525‐nm light (green bar) at baseline (black) and following drug application (cyan). Effects of meclofenamate are seen within 5 min, whereas the effects of carbenoxolone and octanol occur between 20 and 30 min (data plot times).

Finally, in addition to extracellular potassium contributing to the light response, it is possible that some potassium directly flows through gap junctions connecting Müller cells and astrocytes. We observed that a variety of gap junction blocking drugs disrupt the astrocyte light response (Figure 7). Meclofenamate acts quickly in this regard (within 5 min), whereas carbenoxolone and octanol take between 20 and 30 min to noticeably affect the response. Carbenoxolone eliminates the light response like meclofenamate but does not affect RMP stability to the same degree. Octanol was able to reduce the light response but not eliminate it, even after an hour of exposure. The effects of all inhibitors were not reversible within the timeframe studied (30 min–1 h of washout).

## 4 Discussion

Recently, we described heterogeneity in retinal astrocyte morphology at the single‐cell level (Holden et al. 2023, 2024, 2025). In that work, we identified recurring microstructural motifs which were predictive of neuronal and vascular architecture of the inner retina. Currently, the functional significance of each motif is unknown, but the structure and placement of neuronal‐associated motifs (bristle, bead, and pad) lead us to suspect their involvement in monitoring electrical activity in the inner retina or directly affecting neuronal excitability (Holden et al. 2023). The astrocytic bristle motif is reminiscent of dendritic spines in their shape and is itself highly predictive of neuroglial contact. In particular, near the optic nerve head, bristled astrocyte processes cross multiple fascicles of axons and intercalate bristles within the bundles to contact individual axons (Holden et al. 2023). While synapses are not found in the NFL, the structural similarities between bristles and dendritic spines are striking and suggest a similar function in summing neural‐derived ionic currents for integration (Araya et al. 2006; Yuste 2011; Cornejo et al. 1979). Furthermore, other neuronal‐associated astrocyte structures such as the bead and the pad motifs contact axons and also ganglion cell soma near the AIS, which might support modulation of excitability (Holden et al. 2023).

Before beginning to understand the electrophysiological roles of neuronal‐associated astrocyte motifs, we need to better understand retinal astrocyte electrophysiology at a whole‐cell level so we can appreciate any nuance at a regional membrane level. Here, we were primarily interested in whether (1) astrocytes have an electrical response to light like what has been observed in Müller glia and (2) whether mouse IV relationships are similar to those observed in rabbit astrocytes, Müller glia of various species, or astrocytes in other regions of the CNS (Clark 1993; Clark and Mobbs 1994; Newman 1985a; Bordey and Sontheimer 1998; D'Ambrosio et al. 1998; Zhou et al. 2021). We know from the wealth of literature on Müller glia that they exhibit potassium and calcium‐dependent depolarization in response to light (Newman 1985a, 2005; Marchese et al. 2022; Bringmann et al. 2006). This appears to be a conserved response observed across species (Conner et al. 1985; Kline et al. 1985). Additionally, we know that Müller glia are largely permeable to potassium and calcium from studies of their IV relationships and that they respond to the activity of inner retinal neurons (Newman 1985a; Bringmann et al. 2006). We tested whether astrocytes similarly have an electrical response to light and found that they do. Initially, we assumed that the observed depolarization to light onset and offset was due to neuronal‐released potassium in the NFL due to action potential firing. In support of this, astrocytes depolarize to KCl application and have their light response abolished by BaCl_2_. Periodic large magnitude depolarizations are also induced by BaCl_2_ which are not coincident with light onset or offset. We suspect this may be due to the buildup of extracellular potassium with occasional large currents when there is chance dissociation of barium from the potassium channels. Barium is also known to transiently activate BK channels prior to blockade (Zhou et al. 2012). A similar process could help explain our results as well. Furthermore, when current is injected into cells exposed to BaCl_2_, their membrane potential is brought back to baseline levels, but the light response is not restored. This tells us that the lack of a light response is directly due to potassium conductance and not a lack of driving force for potassium to enter the cell. Surprisingly, however, the astrocyte light response was resistant to blockade of neuronal spiking using aTTX, meaning the majority of the light response is not due to potassium originating from action potentials. The concentration of aTTX used was high enough to block Nav1.1–1.4, 1.6–1.7, and alpha subunits 1–3 and 6 have been reported in the ganglion cell layer of the rodent retina (Fjell et al. 1997; Risner et al. 2020; McGrady et al. 2022; Gilchrist et al. 2014). This tells us that aTTX blocks voltage‐gated sodium channels found on the cells of the GCL (RGCs and some amacrine cells). It should be noted, however, that neurons other than RGCs and amacrine cells depolarize to light stimulus and can release potassium. However, such neurons (like bipolar cells) are found deeper in the retina and do not contact astrocytes. It is unlikely that these neurons, whose potassium depolarizes Müller cells, also directly contribute the potassium to depolarize astrocytes. The difference we observe in latency for Müller cells and astrocytes is not significantly different, so would not be in accordance with the time needed for potassium to diffuse from the plexiform layers to the NFL.

It is possible that the presence of different microstructural motifs as described in (Holden et al. 2023) could contribute to heterogeneity in astrocyte electrophysiological response to light (Holden et al. 2023). We did not observe this type of relationship based on the dye‐filling experiments we conducted. However, for morphological studies, the amount of cells we recorded from and filled with dye is low. Additionally, most astrocytes exhibit most neuronal‐associated motifs. With our current data set, we cannot find correlations in motif expression and physiology. This would require either a dramatically increased sample size of recorded cells or a different experimental methodology.

While recording from astrocytes, we occasionally patched a Müller glia endfoot which wrapped around the astrocyte soma. In doing so, we observed that the overall appearance and kinetics of the light response in both glial cell types were very similar. Because Müller glia siphon potassium to the vitreal surface and release it at their endfeet, it would make sense that astrocytes may uptake potassium themselves to help maintain ionic balance, resulting in a similar voltage response (Karwoski et al. 1989; Newman et al. 1979; Kofuji and Newman 2004; Newman 1986). If the potassium which promotes astrocyte depolarization originated primarily in ganglion cell axons, we would expect the depolarization amplitude to vary with retinal eccentricity. Closer to the optic nerve head, where ganglion cell axons converge, the total potassium released per unit area would be greater than in the periphery and cause increased astrocyte depolarization. We do not observe this, and instead depolarization amplitude is invariant with location in the retina. This could be explained if the potassium originated from Müller cells whose density is relatively constant with eccentricity. Because astrocytes do uptake extracellular potassium, however, it also suggests that Müller endfeet are crucial in buffering this difference in potassium concentration. In the literature, Müller glia RMP is often more hyperpolarized than −80 mV, and the light‐induced depolarization can exceed 1 mV. Any differences in our reported electrophysiology (RMP or depolarization magnitude) are likely due to experimental differences in bath solution potassium concentration, the use of in situ vs. dissociated cells, species‐specific differences, and wavelength of stimulation. We found that in papers showing Müller cell light‐induced depolarization, bath solutions varied in ionic composition, particularly in potassium concentration (ranging from 2.5 mM to 5 mM). The reported ranges of potassium concentration alone could account for around 9 mV of RMP difference across papers (Reichelt et al. 1993; Pannicke et al. 2017; Felmy et al. 2001; Zhao et al. 2012; Chao et al. 1997; Kofuji et al. 2000; Francke et al. 2001; Bringmann et al. 1999). Moreover, in the literature there are recordings from cells both in situ and while dissociated from the retina which may add variability to the reported metrics. We include Figures S1 and S2 to verify the health of recorded cells and the stability of the patch. In S1, we show differences in glial cell depolarization when stimulated with green vs. blue light. There was significant variability in the response amplitudes (sometimes as much as 2‐3X as shown in the figure). Published literature may use wavelengths of light that better stimulate retinal glia compared to our use of 525 nm for patch experiments. Our choice of 525 nm was a technical one as we needed to use it to find tdTomato‐positive cells and aid in achieving a successful patch. Switching filter cubes to allow blue light stimulation induced vibration that often caused a loss of the patch. Moreover, we were able to record from patched cells for over an hour without a significant depolarization in cell RMP. In S2, we show an example cell recorded from an hour after baseline. Instead of depolarizing (as would be expected if the cell was dying), it actually hyperpolarized by a few millivolts, likely due to an improved seal over time. The amplitude of response to light was the same at both time points.

Additional similarities in astrocyte and Müller electrophysiology were observed while recording current flux in response to voltage step. We observed both inward and outward rectification, in addition to cells which passed low current at all potentials. The outward rectification and a portion of the inward rectification responses were very similar to rabbit astrocytes and ex vivo Müller recordings from salamander (Clark 1993; Clark and Mobbs 1994; Newman 1985a). These similarities suggest that most of the ionic conductance of both mouse retinal astrocytes and Müller glia is due to potassium and calcium and supports the idea of a potassium‐mediated light response. However, we were unable to find recordings in the literature to completely describe the inwardly rectifying response. A portion of the response is likely due to calcium or sodium currents, but we observe a linear negative IV relationship far into the positive applied potentials which cannot be described by the sodium‐specific IV curve reported in Clark and Mobbs 1994 or the calcium‐specific IV curve in Newman 1985. It is possible that the IV trace in Figure 4G represents a single subtype of calcium or sodium channel and that others exist with a similar yet shifted curve. If multiple subtypes exist on the glial cells, then perhaps the currents would sum together to create the emergent relationships in Figure 4A. Single‐cell RNA‐sequencing studies to parse the differences in gene expression between Müller glia and astrocytes are necessary, perhaps using the recently developed astrocyte‐specific Pax8‐driven Cre mice (Cullen et al. 2024). To our knowledge, no scRNA sequencing dataset exists, which focuses on astrocytes. The existing datasets are whole‐retina, and due to the low proportion of astrocytes, very few of them are represented in each dataset (and never enough for proper clustering and statistics). Because Pax8 is a selective marker for astrocytes, it could drive the expression of a fluorescent marker, which could be used in flow cytometry sorting prior to single‐cell sequencing to enrich for astrocytes alone. We were also surprised to see that repeated acquisition of the IV curve data caused the relationship to change in both astrocytes and Müller glia. Cells with initial outward rectification can decay to a flat IV relationship and be made to flip polarity to inward rectification. The opposite is true for cells with initial inward rectification. Mechanistically, we do not yet know why this occurs. It is possible that there is a stimulus‐dependent change in cell conductance, possibly through channel inactivation or internalization. It is also possible that repeated stimulation depletes the cells' energy reserves as it moves to re‐establish ionic gradients. This might affect Na^+^/K^+^ ATPase activity, altering ionic distribution and thus the IV relationship. Because repeated stimulation can flip IV relationship polarity, we suspect that our recordings of IV relationships simply capture cells in a probabilistic state of outward or inward rectification (or low conductance) rather than being suggestive of subtypes of each cell class. Because IV relationships inform us about ionic currents, observing that both macroglial cell types have the same IV curves strengthens the case that the light response is due to the same currents in astrocytes as in Müller glia (potassium).

Because of reports of light‐induced calcium responses in rat, guinea pig, and avian Müller glia, we wanted to verify that the mouse astrocyte depolarization to light was not a calcium response (Newman 2005; Rillich et al. 2009; Marchese et al. 2022). In rat and guinea pig, fast calcium transients are likely due to purinergic signaling causing the release of internal calcium stores. Astrocytes express purinergic receptors so could theoretically undergo a similar process (Rillich et al. 2009; Newman 2001). We did not find evidence for light‐induced calcium responses in astrocytes, which is consistent with previous reports in rats (Newman 2005). However, the degree to which we observed light‐induced calcium responses in Müller glia was far less than expected. This may be a species‐specific difference between mouse and rat, given the same protocol was used as previous reports (Newman 2005). Regardless, because some Müller glia did have transient light‐induced calcium elevation, if the astrocyte light response were due to calcium we would expect to be able to observe it. This is because the light‐induced depolarizations we recorded were similar in magnitude between Müller endfeet and astrocytes, so it is unlikely that a detection limit of the Fluo4‐AM could explain our failure to observe it (and the detection limit is very low) (Paredes et al. 2008). It is possible that calcium transients occur in astrocytes at a sub‐threshold level, but they would not explain our observed light responses. In Figure 5C,F, there is a linear decrease in fluorescence which is due to photobleaching. If there were a calcium‐based light response in astrocytes, there would be a deviation from this linearity as is seen with the Müller cells. The lack of a light‐induced calcium response in astrocytes is curious, given the observation that mechanical stimulation of the retina causes a calcium response and associated modulation of neural activity (Newman and Zahs 1998). However, it is likely that to induce this phenomenon physiologically, a more specialized stimulus is needed rather than a full‐field narrow band light source. It is also possible that a wavelength‐specific response occurs with respect to calcium signaling, as different wavelengths of light were used in patch clamp vs. calcium imaging experiments. It should be noted that the green light used in the patch clamp experiments is closer to the peak sensitivity of mouse rod photoreceptors than the blue light used for the calcium experiments.

**FIGURE 8:**
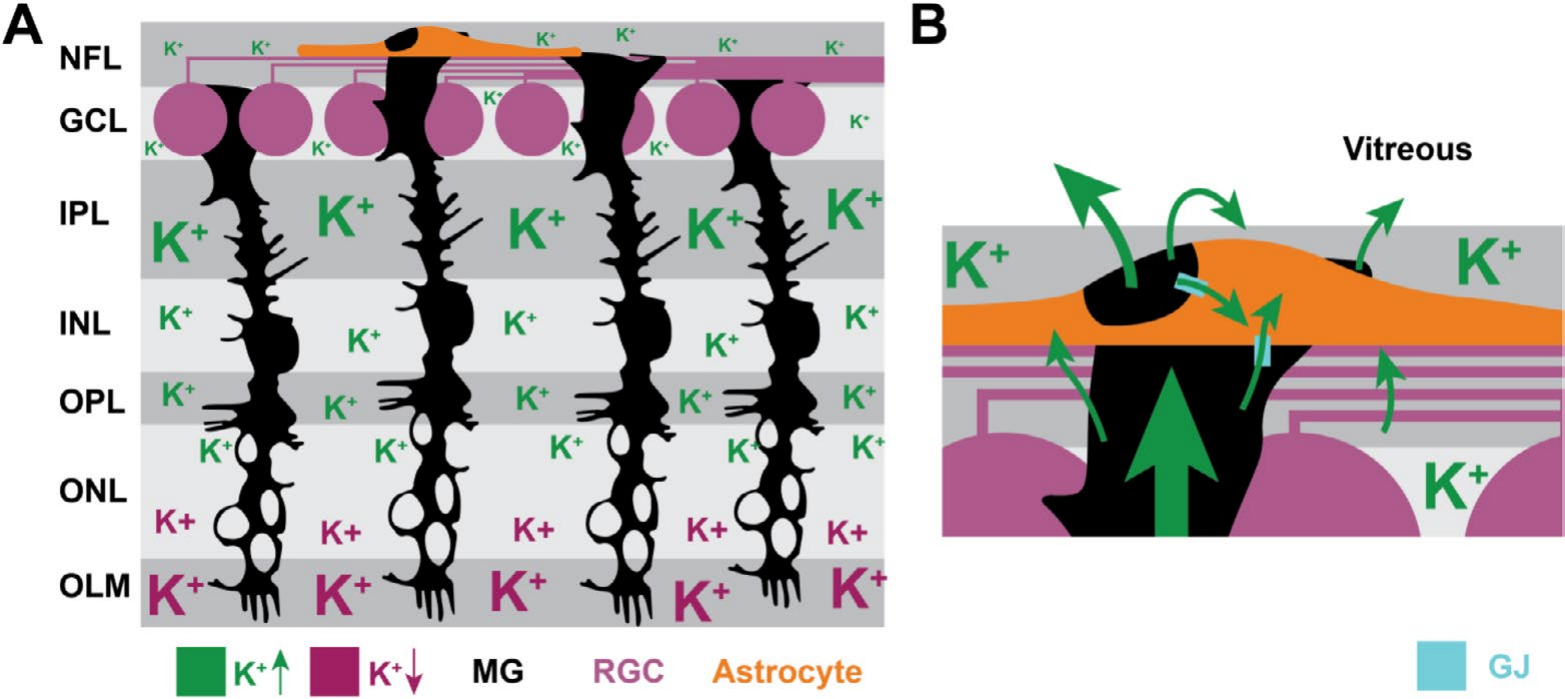
Model of astrocyte light response. Retinal cross section (A) and zoomed in (B) view of a potassium‐based model of astrocyte depolarization to light. Most of the potassium arises from Müller glia siphoning of potassium to the inner retina. This potassium is released extracellularly and through gap junction connections with astrocytes. Extracellular potassium released at focal sites of endfoot association with astrocyte cell bodies depolarizes the cells. A small amount of potassium released from RGC axons also contributes to astrocyte depolarization.

Using the relative latency to respond to light for astrocytes and ON RGCs, we modeled potassium release and diffusion from axons and how it would affect astrocyte potential. We similarly used the latency difference between astrocytes and Müller glial responses to estimate the number of Müller glia which could contribute potassium to the astrocyte light response. This modeling supports a dual origin of potassium which is consistent with the aTTX recordings. For a small number of axons (~10), the degree of astrocyte depolarization is consistent with recordings. This is a reasonable assumption for the number of axons that could cross across an astrocyte's domain based on direct observation of RGC cell density (2500 cells/mm^2^) and quantifying average astrocyte area (2407 μm^2^, 8180 μm^2^ for convex hull) (Holden et al. 2023). The number of RGCs within the potassium diffusion‐defined radius of 9.5 μm from the astrocyte soma edge is closer to 4 RGCs on average based on direct observation of astrocyte immunolabeling (Holden et al. 2023). However, the model fails to accurately predict astrocyte depolarization at axon bundles, which contain hundreds of axons—likely highlighting a significant role of Müller glia endfeet in buffering potassium there. With potassium diffusion modeling, we estimate that between 4 and 5 Müller cells could release potassium which each astrocyte responds to. In the literature, ex vivo recordings at salamander Müller endfeet show potassium concentration increases of ~0.1 mM in response to local KCl stimulation (Newman et al. 1979), and our GHK modeling shows that creating the astrocyte light response requires an elevation of 0.19±0.01 mM K^+^. It is reasonable that 4–5 Müller cells could release potassium at a localized endfoot concentration of ~0.1 mM and through diffusion generate an overall potassium elevation of ~0.19 mM. However, because the difference in depolarization latency between astrocytes and Müller glia is non‐significant, it is likely that the diffusion time is less than approximated, and 4–5 is an overestimate of the number of Müller cells which contribute potassium to the astrocyte response. It is more likely that only 1–2 Müller glia release potassium directly onto the astrocyte surface at a high local concentration (we observed many astrocytes with Müller endfeet ensheathing the cell body). In support of this, measurements of potassium levels across retinal layers in response to light shows that potassium increases in the IPL are around an order of magnitude greater than at the NFL (Dick et al. 1985; Karwoski and Proenza 1978). Despite this, our recordings do not show such a large disparity in depolarization amplitude between astrocytes and Müller glia. This could be reconciled if the potassium which depolarizes astrocytes is siphoned directly through Müller glia from the IPL, thereby depolarizing each to a similar degree. A small loss in current during transfer could explain why the Müller ON response is slightly larger in amplitude than the corresponding astrocyte response. Because the potassium released at Müller endfeet arises from a siphoning process, the quantity of potassium entering the cell is equal to that released at the endfoot (Newman et al. 1979). In amphibian species, the light‐induced increase in potassium concentration in the inner plexiform layer due to 3 mM light (similar to our full‐field illumination experiments) is between 0.18 mM (frog) and 0.2 mM (mudpuppy), precisely the concentration needed to replicate the light response we see in mice (Karwoski and Proenza 1978). Additionally, the short latency difference between Müller and astrocyte responses means it is unlikely that astrocytes depolarize to potassium which diffuses from the IPL directly through the extracellular space. These data support a model where the same potassium which depolarizes Müller glia is siphoned and released directly onto astrocytes. We propose a model where the majority of the astrocyte light response is due to focal release of potassium from Müller endfeet onto the astrocyte soma, with a smaller contribution of potassium originating from RGC axons (Figure 8).

It is possible that the identity of the ganglion cells directly beneath an astrocyte is partially responsible for its electrical response profile to light. In this study, we did not determine the identity of the ganglion cells in contact with the astrocytes because our recording setup cannot accommodate dual patch recordings. Additionally, patching RGCs directly beneath the astrocyte would require perforating the astrocyte with the pipette tip, which would interfere with recovering morphology or obtaining a healthy recording.

We also observed a sensitivity in the light response to the gap junction inhibitors carbenoxolone, meclofenamate, and octanol. It is possible that some potassium directly flows between Müller glia and astrocytes through gap junctions (Robinson et al. 1993; Zahs and Newman 1997). We observed a difference in the efficacy of the light response blockade by each inhibitor. The difference in performance is likely due to a combination of factors including (1) different time scales and kinetics of binding, (2) binding efficiencies for gap junctions and their subsequent ability to block material flux, and (3) differences in off‐target binding sites. All drugs are consistent in blocking the light response in some capacity and in some time frame. The effect of meclofenamate addition is most pronounced (complete abolishment of light response), and the onset is more rapid than either carbenoxolone or octanol (~5 min vs. 20–30 min). The effect of meclofenamate persists throughout the 30–60 min we recorded from cells but is presented at the 5 min timepoint to highlight differences in effect time. This is interesting given meclofenamate's greater capacity to bind potassium channels (Manjarrez‐Marmolejo and Franco‐Pérez 2016). Because we propose the light response is due to potassium flux, this is not entirely surprising. It should be noted that carbenoxolone can be toxic to the retina, which could contribute to its apparent block of the light response (Pan et al. 2007). We interpret the decrement in light response in the presence of the gap junction inhibitors as evidence that at least some of the Müller‐derived potassium may directly flow into astrocytes through gap junctions. It does not say this is the exclusive route that potassium takes, and likely not even the most common.

Gap junction blocking drugs are relatively non‐selective and bind to voltage‐gated potassium and calcium channels, which are of interest in the light response (Manjarrez‐Marmolejo and Franco‐Pérez 2016; Pan et al. 2007; Vessey et al. 2004). Additionally, they block upstream circuitry including horizontal and amacrine cells (Pan et al. 2007; Veruki and Hartveit 2009). We include the data because a natural question which arises from our experiments is whether the potassium currents flow through gap junctions, and others may be tempted to run these experiments. It is also important to note that gap junction inhibitors will also block connexin hemichannels. It is possible that fast calcium transients in Müller cell end feet cause hemichannel‐mediated release of gliotransmitters, which could stimulate astrocytes to uptake potassium or depolarize in another way. Because our recordings supply the inhibitors with the bath solution, this process could be blocked. While our data is clear that the light response is due to potassium, the route the potassium takes to enter the astrocyte is not certain. Additionally, the potassium depolarization could be working in concert with an independent mechanism of gliotransmitter‐motivated depolarization originating with Müller cells.

This work fills a critical gap in our understanding of retinal astrocyte electrophysiology and shows both similarities and differences with Müller cells. This baseline understanding of astrocyte electrophysiology will be useful as we look for functional specialization in membranous astrocyte microstructures that could influence neuronal–vascular coupling in the retina.

## Author Contributions

J.M.H. conducted the experiments, analyzed the data, and wrote the manuscript. A.M.B. recorded naïve α‐ON RGC recordings. D.J.C. and L.K.W. provided funding and other resources, supervision, project management, and manuscript editing.

## Conflicts of Interest

The authors declare no conflicts of interest.

## Supporting information


**Figure S1.** An example astrocyte stimulated with light at two different wavelengths (525 nm green vs. 460 nm blue). The responses varied in amplitude and the time frame to recover from stimulation.

**Figure S2.** Recorded cell RMP remains stable over time. After an hour, RMP remains within 3.5 mV of baseline and drifts in the hyperpolarizing direction in this example not depolarizing. The amplitude of response an hour into the recording is the same as at baseline.

## Data Availability

All data supporting the conclusions of this manuscript will be made available upon reasonable request to the corresponding author.
