# Supplementary figures and images for: Potassium‐Dependent Coupling of Retinal Astrocyte Light Response to Müller Glia
Source: Glia. 2025 Apr 22;73(7):1520–34. doi: 10.1002/glia.70022 (PMC12121466; doi:10.1002/glia.70022)

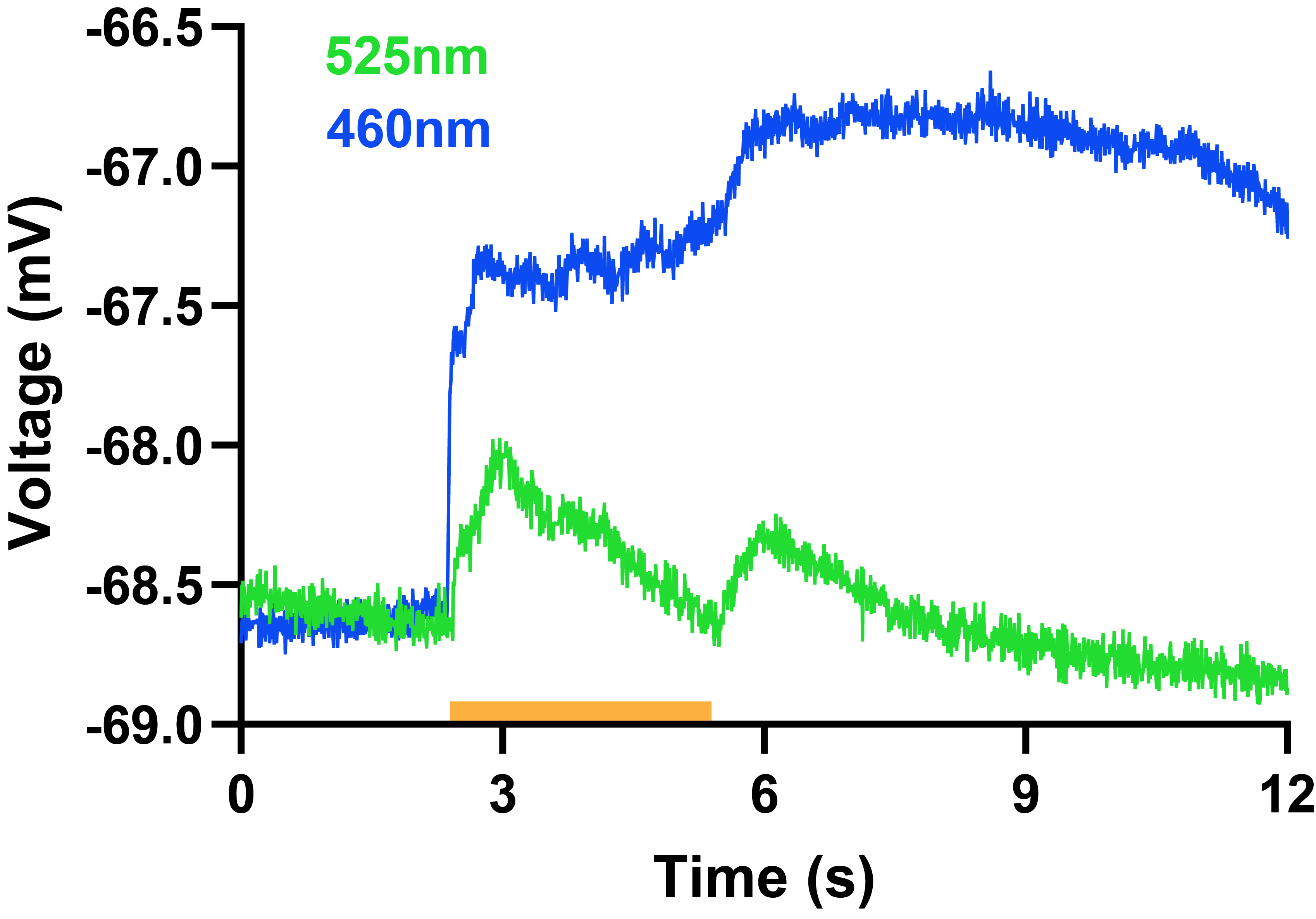

Supplement: Supplementary file 1 — Figure S1. An example astrocyte stimulated with light at two different wavelengths (525 nm green vs. 460 nm blue). The responses varied in amplitude and the time frame to recover from stimulation. [file GLIA-73-1520-s002.png]

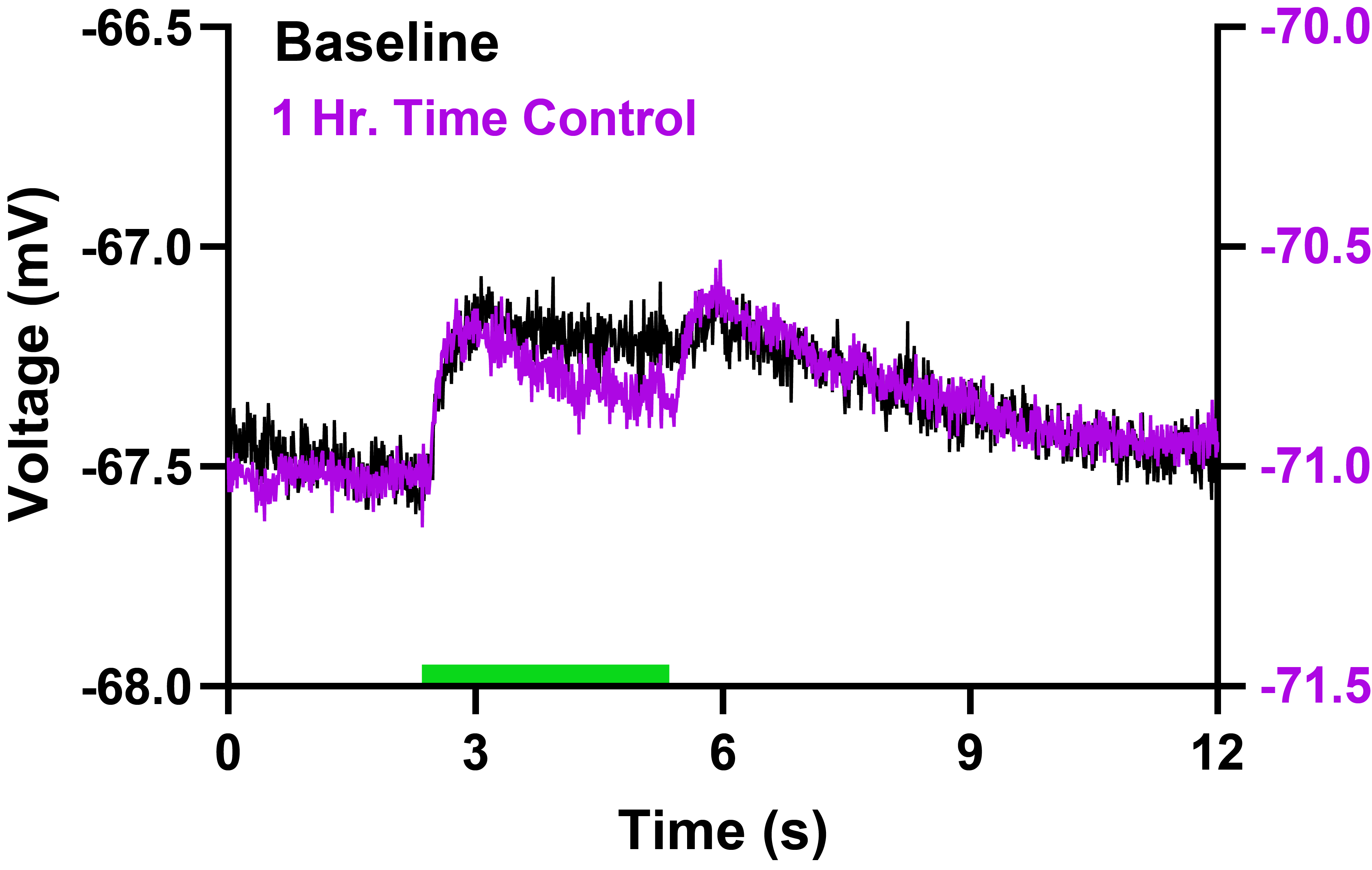

Supplement: Supplementary file 2 — Figure S2. Recorded cell RMP remains stable over time. After an hour, RMP remains within 3.5 mV of baseline and drifts in the hyperpolarizing direction in this example not depolarizing. The amplitude of response an hour into the recording is the same as at baseline. [file GLIA-73-1520-s001.png]
